# Tirzepatide Versus Semaglutide on Weight Loss in Type 2 Diabetes Patients: A Systematic Review and Meta‐Analysis of Direct Comparative Studies

**DOI:** 10.1002/edm2.70045

**Published:** 2025-04-04

**Authors:** Jimmy Wen, Burhaan Syed, Denise Nadora, Christiane How‐Volkman, Ethan Bernstein, Alina Truong, Muzammil Akhtar, Adam Razick, Jose Puglisi, Eldo Frezza

**Affiliations:** ^1^ California Northstate University College of Medicine Elk Grove California USA; ^2^ University of California, Los Angeles Los Angeles California USA

**Keywords:** diabetes, meta‐analysis, semaglutide, tirzepatide, weight loss

## Abstract

**Introduction:**

Glucagon‐like peptide‐1 receptor agonists (GLP‐1RAs) have emerged as an efficacious treatment for type 2 diabetes mellitus (T2DM) and have demonstrated substantial weight loss effects. This systematic review compares two prevalent GLP‐1RAs, tirzepatide and semaglutide, with their weight loss effects and rates of adverse events (AEs).

**Methods:**

Following the Preferred Reporting Items for Systematic Reviews and Meta‐Analyses (PRISMA), a systematic search was performed in PubMed, Embase and Cochrane Library for direct comparative studies between tirzepatide and semaglutide. A meta‐analysis was conducted via a random‐effects model to analyse the differences in weight loss outcomes between study cohorts.

**Results:**

Four studies, with 28,827 patients (14,870 tirzepatide/13,928 semaglutide), mean age of 55.7 years (52.0 to 63.7) and mean follow‐up of 35.9 weeks (23.6 to 44.6), were included in this study. Mean weight change across four studies for tirzepatide and semaglutide was −11.4% (−15.3% to −8.27%) and −7.3% (−8.3% to −6.08%), respectively. The meta‐analysis supports these findings with a mean difference of −4.84 kg (95% CI: −6.21 to −3.47), favouring tirzepatide. The most common AEs were minor and moderate‐severity gastrointestinal (GI) AEs.

**Conclusion:**

Current literature supports tirzepatide demonstrating a higher impact on weight loss than semaglutide, with both demonstrating high rates of minimal‐ to moderate‐severity AEs. Further research with comparative head‐to‐head trials will better elucidate these weight loss effects and safety profiles.

## Graphical abstract

The aim of this systematic review and meta‐analysis was to synthesise the research on direct comparative studies between tirzepatide and semaglutide on weight loss among patients with type 2 diabetes. Four studies were included, and a random‐effects model meta‐analysis was conducted on estimated treatment differences, favouring tirzepatide over semaglutide.

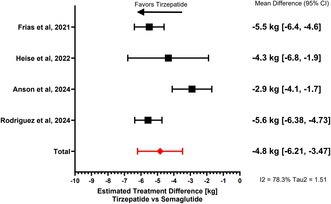

## 1 Introduction

Obesity is a chronic disease and is considered a global epidemic, with high associations with but not limited to cardiovascular diseases, type 2 diabetes mellitus (T2DM), several malignancies, chronic kidney disease, metabolic dysfunction‐associated steatosis liver disease (MASLD) and mental disorders [1]. Thus, the prevention and treatment of obesity are important to improve healthcare outcomes, quality of life and decrease overall healthcare costs. Currently, treatment for overweight and obesity includes healthy lifestyle changes, behavioural counselling, prescription medications and surgery [2]. Given its detrimental effects on individual health and its widespread impact on society, there is great interest in the development of therapeutics to combat obesity and T2DM and their associated sequelae.

Glucagon‐like peptide‐1 receptor agonists (GLP‐1RAs) have emerged as a prominent treatment for T2DM with a notable weight loss effect via delayed gastric emptying, glucagon inhibition and stimulation of central receptors that modulate appetite suppression and energy expenditure [3, 4]. Current Food and Drug Administration (FDA) approved GLP‐1RAs are for glycaemic control (dulaglutide, exenatide, liraglutide, lixisenatide/insulin, semaglutide) or weight loss (semaglutide, liraglutide) [3]. However, tirzepatide, a recent agent that has gained popularity, has shown similar, if not greater, glycaemic control and weight loss in patients with obesity or T2DM [5]. Its mechanism of action involves dual agonism of the GLP‐1 and glucose‐dependent insulinotropic polypeptide (GIP) receptors [6].

Through a comparative analysis, this systematic review aims to compare the efficacy of tirzepatide and semaglutide in facilitating weight loss and their respective safety profiles. A more accurate comparison can be performed through a comparative analysis by reducing the confounding and bias observed in differing patient demographics and study characteristics associated with indirect comparisons or network meta‐analyses. We hypothesise that tirzepatide, due to its dual agonism, will produce greater weight loss with a similar or improved safety profile.

## 2 Methods

### 2.1 Search Strategy

A systematic search following guidelines established by the Preferred Reporting Items for Systematic Reviews and Meta‐Analyses (PRISMA) was performed in PubMed, Embase and Cochrane Library for studies until December 8, 2024. The keywords (((semaglutide) AND (tirzepatide)) AND (weight loss)) AND ((outcomes) OR (efficacy)) were used to perform the systematic review search.

A Patient, Intervention, Comparison, Outcome, Time (PICOT) method was used to formulate the search strategy. The patient population included adult type 2 diabetic patients > 18 years of age. The intervention was tirzepatide or semaglutide treatment in this population. Only comparative studies were included if they directly compared these two drugs. The outcomes in this study were changes in body mass index (BMI), body weight, body composition, reported pre‐ and post‐intervention outcomes and rates of complications. There were no restrictions on the follow‐up period. The inclusion criteria included: (1) comparative studies such as randomised controlled trials (RCTs), cohort studies, case–control studies, real‐world evidence studies investigating tirzepatide versus semaglutide; (2) reporting post‐intervention outcomes such as but not limited to weight loss, BMI and other relevant laboratory values or patient‐reported outcomes. Case reports, review articles, expert opinions, non‐English studies and articles without pre‐ and post‐intervention outcomes reported were excluded. Two reviewers independently reviewed all articles, and any discrepancies were resolved by rigorous re‐review, or a third reviewer was consulted to determine final article inclusion or exclusion. A rigorous reference search was performed for all included studies to determine if there were additional studies to include. This protocol is registered in the PROSPERO database under the registration number CRD42023469616.

### 2.2 Study Quality Assessment

The study's quality and risk of bias (ROB) were assessed utilising the Methodological Index for Non‐randomised studies (MINORS) for non‐RCTs [7] and the Cochrane Risk of Bias tool [8] for RCTs. For MINORS, scores were reported as 0 (not reported), 1 (reported but inadequate), or 2 (Reported and adequate) with a maximum score of 24 for comparative studies. A MINORS score of 21 to 24 was determined to have a low ROB, 16 to 20 was a moderate ROB, and 0 to 16 was a high ROB. Seven domains were analysed with this tool: Sequence generation, Allocation concealment, Blinding of participants and personnel, Blinding of outcome assessors, Incomplete outcome data, Selective outcome reporting and Other sources of bias. These domains were categorised as ‘high’, ‘low’, or ‘unclear’ risk of bias. Each article was double‐blinded and dual‐screened by two independent reviewers. Discrepancies were resolved by re‐reviewing the articles or consulting a third reviewer until a consensus was reached.

### 2.3 Data Extraction and Analysis

Data extraction included study characteristics and variables such as study year, Number of patients, Dosage of intervention, Mean age, Mean follow‐up, Pre‐ and post‐operative reported outcomes and Complications. Mean, percentage, standard deviations, ranges and other descriptive statistics were reported if available and applicable. All data was stored and analysed using Google Sheets (Google Drive; Google, Mountain View, CA). A meta‐analysis was performed using SPSS version 29 (IBM Corp., Armonk, NY, USA) to estimate the pooled effect of weight reduction across the treatments using a random‐effects model. The effect size for each treatment was the mean weight change (in kg) accompanied by a 95% confidence interval (CI). The random‐effects model was chosen to account for potential heterogeneity between studies. The weighted mean effect was calculated by combining individual study effect sizes using inverse‐variance weights adjusted for between‐study variance. Tau^2^, which quantifies the variability among the effect sizes beyond sampling error, was estimated using the DerSimonian and Laird method. The heterogeneity across studies was assessed using the I^2^ statistic, representing the proportion of total variation attributable to heterogeneity. Forest plots were created using GraphPad Prism version 10.

## 3 Results

**FIGURE 1:**
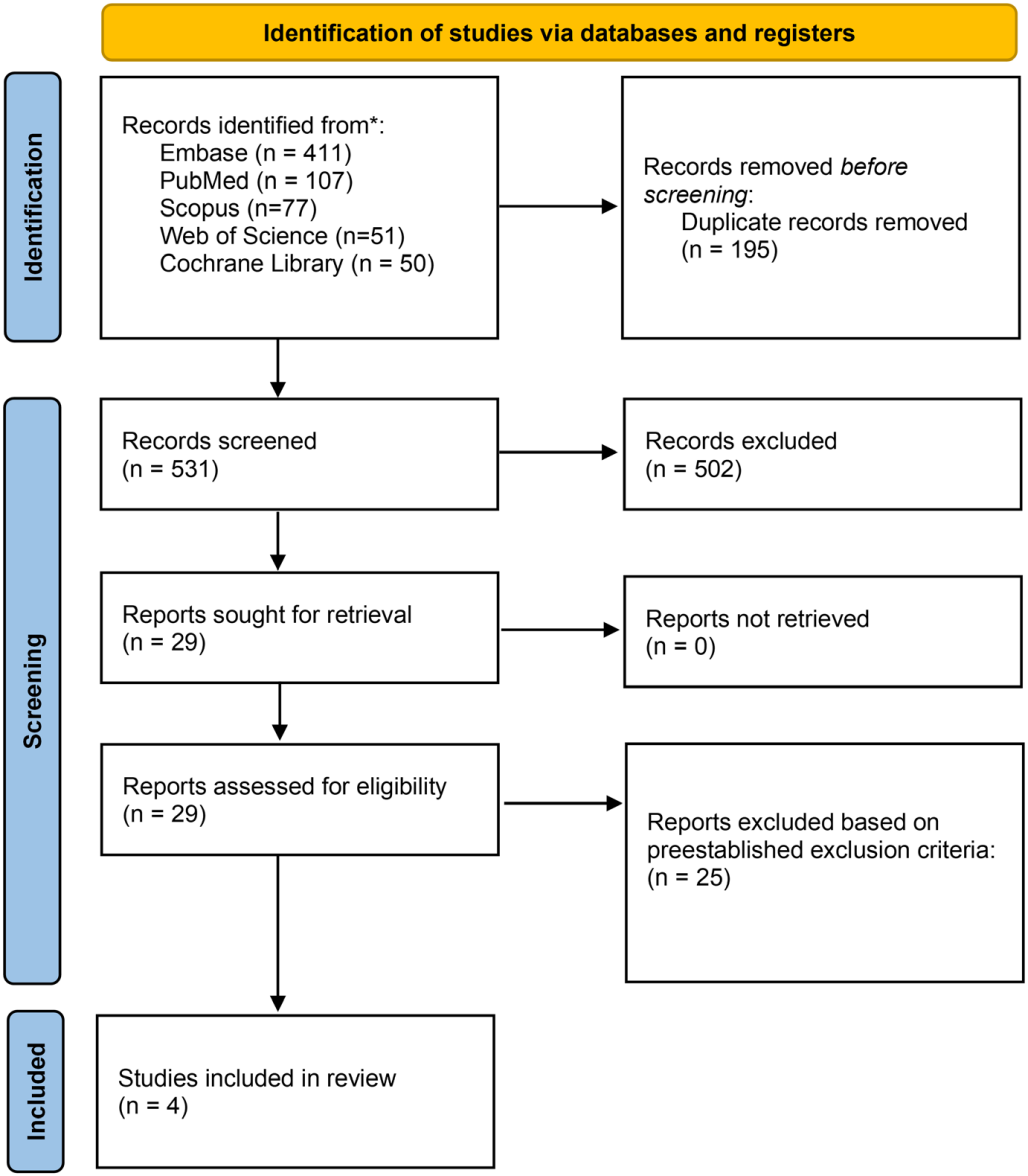
PRISMA diagram of article selection process.

The initial systematic search resulted in 696 studies through PubMed, Embase, Scopus, Web of Science and Cochrane Library. After removing 195 duplicates, 531 studies moved on to the title and abstract screening. Twenty‐nine relevant studies moved on to full‐text screening, after which four remained for this study. The full screening process can be found in Figure 1.

### 3.1 Study Characteristics and Demographic Data

Across the four studies, there were 28,827 T2DM patients (33.9% male, 66.1% female), with a mean age of 55.7 (52.0 to 63.7) years. There were 14,870 patients taking tirzepatide (mean age: 5), 13,928 patients taking semaglutide (mean age: 54.82) and 28 taking a placebo. The mean follow‐up time was 35.9 (23.6 to 44.6) weeks, with mean BMI ranging between 30.8 and 39.1. There were two RCTs [9, 10] and two retrospective observational studies [5, 11]. Tirzepatide dosages ranged from 2.5 mg to 15 mg, and semaglutide ranged from 0.5 mg to 1 mg. One study also included a non‐T2DM cohort, with 13,846 participants (27% male, 73% female) [11]. The patient and study characteristics are summarised in Table 1.

**TABLE 1:** Patient demographics and study characteristics of included studies.

Author	Journal	Study year	LOE	Number of patients (M/F)	Age (years ± standard deviation)	Body mass index (kg/m^2^) (SD)	Follow‐up (weeks)
Frias 2021	The New England Journal of Medicine	2019–2021	2	Tirzepatide 5 mg: 205/265 Tirzepatide 10 mg: 238/231 Tirzepatide 15 mg: 214/256 Semaglutide 1 mg: 225/244	Tirzepatide 5 mg: 56.3 ± 10.0 Tirzepatide 10 mg: 57.2 ± 10.5 Tirzepatide 15 mg: 55.9 ± 10.4 Semaglutide 1 mg: 56.9 ± 10.8	Tirzepatide 5 mg: 33.8 ± 6.85 Tirzepatide 10 mg: 34.3 ± 6.60 Tirzepatide 15 mg: 34.5 ± 7.11 Semaglutide 1 mg: 34.2 ± 7.15	40
Heise 2022	Lancent Diabetes Endocrinol	2019–2021	2	Tirzepatide 15 mg: 31/14 Semaglutide 1 mg: 34/10 Placebo: 21/7	Tirzepatide 15 mg: 61.1 (7.1) Semaglutide 1 mg: 63.7 (5.9) Placebo: 60.4 (7.6)	Tirzepatide: 31.3 (5.01) Semaglutide: 30.8 (3.84) Placebo: 32.2 (3.96)	28
Rodriguez 2024	JAMA Internal Medicine	2022–2024	3	Tirzepatide: 2707/6484 Semaglutide: 2707/6486	Tirzepatide: 51.9 (12.7) Semaglutide: 52.0 (13.2)	Tirzepatide: 39.0 ± 8.1 Semaglutide: 39.1 ± 8.1	23.6
Anson 2024	EClinical Medicine	2023–2024	3	Non‐T2DM Tirzepatide: 1869/5054 Semaglutide: 1869/5054 T2DM Tirzepatide: 1689/2534 Semaglutide: 1689/2534	Non‐T2DM Tirzepatide: 47.5 ± 11.8 Semaglutide: 47.5 ± 11.9 T2DM Tirzepatide: 53.9 ± 10.7 Semaglutide: 54.0 ± 12.2	NR	Tirzepatide: 43.1 Semaglutide: 44.6

Cochrane ROB and MINORS were used, as there were two RCTs and two RCTs. For Cochrane, the two RCTs generally showed a low risk of bias for most of the domains. Frias et al. showed a high risk of bias for the blinding of participants and personnel, as well as having other potential sources of bias. Heise et al. showed a low risk of bias in all domains. The Cochrane risk of bias scores are summarised in Table S1. As for MINORS, the two RCTs had a score of 20–24, while the two RCTs had a score of 15–19. Thus the ROB was determined to be moderate in two studies and low in two studies. The MINORS scores are summarised in Table S2.

### 3.2 Weight Loss

Changes in body weight and fat mass were measured across all four studies, with 3 studies reporting on T2DM demographic [5, 9, 10, 11], while 1 study reported on patients with and without T2DM [11]. The mean weight change across the three studies with a T2DM demographic for tirzepatide was −11.4% (−15.3% to −8.2%), while for semaglutide it was −7.3% (−8.3% to −6.08%) [5, 9, 10, 11]. A full summary of weight loss parameters is provided in Table 2.

**TABLE 2:** Weight loss effects of tirzepatide and semaglutide.

Author	Tirzepatide dose	Outcomes	Pre‐tirzepatide intervention, kg (SD)	Post‐tirzepatide intervention, kg (SD)	% Weight Change	Semaglutide dose	Pre‐semaglutide intervention, kg (SD)	Post‐semaglutide intervention, kg (SD)	% Weight change	*p*
Frias 2021	5 mg	Total body weight	92.5 (21.76)	84.9	8.22	1 mg	93.7 (21.12)	88	6.08	NR
	10 mg		94.8 (22.71)	85.5	9.81					
	15 mg		93.8 (21.83)	82.6	11.94					
Heise 2022	15 mg	Total body weight	94.2 (14.0)	83.0	11.89	1 mg	92.7 (14.0)	85.8	7.44	< 0.001^a^
		Fat mass	36.8 (11.5)	27.1	26.5		35.3 (8.0)	29.4	16.1	0.001^a^
		Fat‐free mass	57.7 (9.3)	55.9	2.8		56.3 (10.3)	55.5	1.4	0.018^a^
Rodriguez 2024	5 mg	Total body weight	110 (25.7)	93.17	15.3%	0.5 mg	110 (25.8)	100.87	8.3%	NR
Anson 2024	NR	Total body weight	99.9 ± 25.8	92.2	7.7%	NR	101.4 ± 24.3	96.6	4.7%	Tirzepatide: < 0.001 Semaglutide: < 0.001

^a^ Between comparators.

**FIGURE 2:**
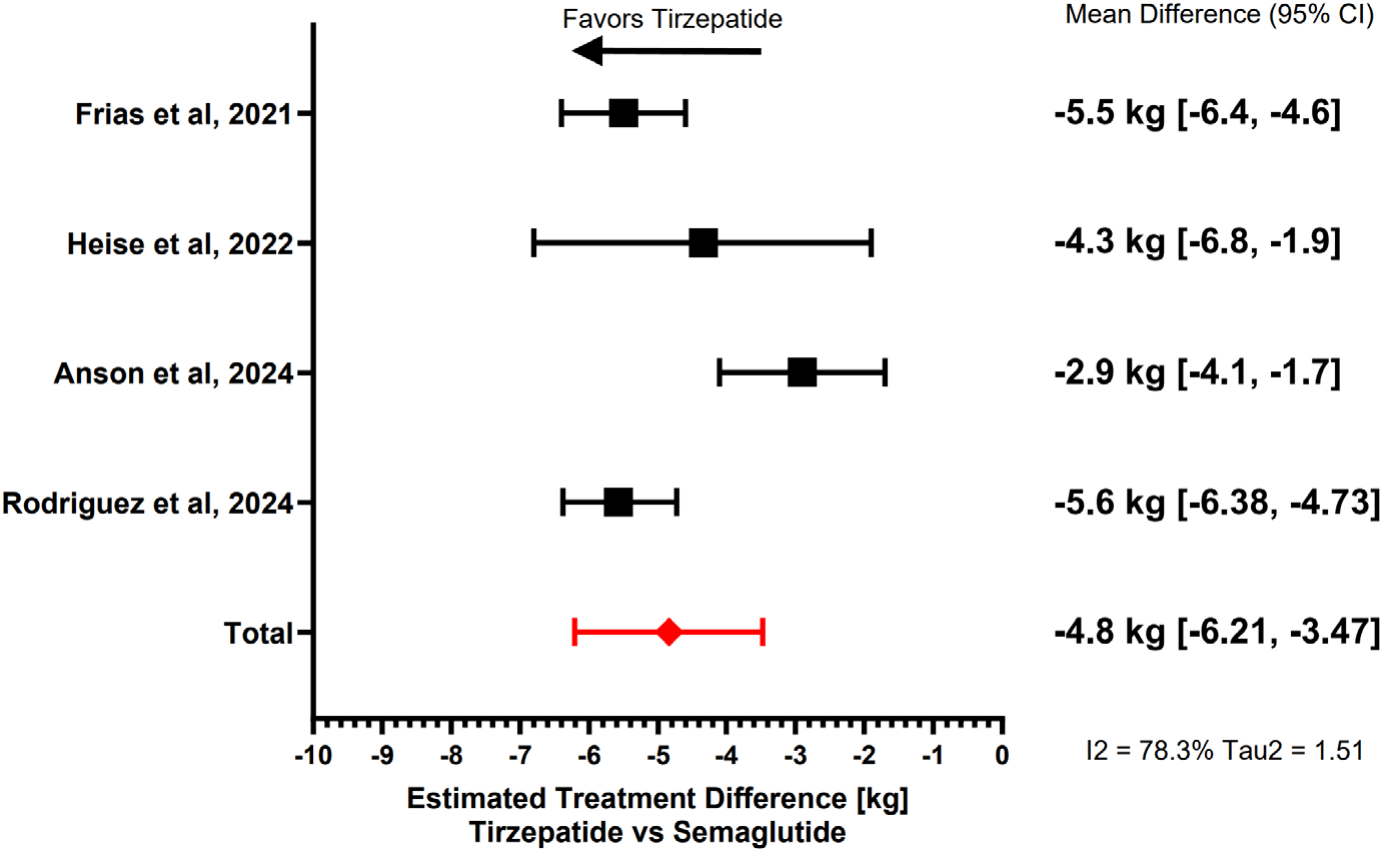
Estimated treatment differences in weight loss between tirzepatide and semaglutide.

A meta‐analysis between tirzepatide and semaglutide was conducted, and the estimated treatment difference (kg) favoured tirzepatide over semaglutide across four studies [5, 9, 10, 12]. The overall treatment effect was calculated using the Random‐Effect model and resulted in a mean difference of −4.84 kg (95% CI: −6.21 to −3.47). The *I* ^2^ statistic, which quantifies heterogeneity, was 78.3% and Tau2, a measure of between‐study variance, was 1.51. The individual study estimates range from −2.9 kg to −5.6 kg, showing consistent superiority of tirzepatide in weight reduction compared to semaglutide across different trials, although the moderate‐to‐high variability of the data expressed by I^2^ and Tau^2^ should make us cautious in interpreting pooled results. The forest plot can be seen in Figure 2.

In Frias et al., a max dose of tirzepatide (5, 10, 15 mg) and semaglutide (1 mg) was compared in a total of 1878 patients, with 1409 patients in the tirzepatide group and 469 patients in the semaglutide group [9]. A significant decrease in weight was observed at the end of the 40 weeks across all groups (*p* < 0.001). All tirzepatide groups were found to significantly decrease weight greater than semaglutide, in a dose‐dependent relationship. Estimated treatment differences with 5 mg, 10 mg and 15 mg of tirzepatide were −1.9 kg (95% CI: −2.8 to 1.0), −3.6 kg (95% CI: −4.5 to −2.7) and − 5.5 kg (−6.4 to −4.6) compared to semaglutide (*p* < 0.001). This was also seen with the proportion of patients achieving 5%, 10% and 15% of body weight (using treatment‐regimen estimand), with tirzepatide having 65%–80%, 34%–57% and 15%–36% achieving these endpoints compared to 54%, 24% and 8% of semaglutide patients, respectively [9].

In Heise et al. (*n* = 117), a max dose of tirzepatide (15 mg/day), semaglutide (1 mg/day) and placebo groups were evaluated over 28 weeks [10]. It was found that the tirzepatide group had a significantly greater decrease in weight compared to semaglutide and the placebo group at both weeks 5 and 28 (*p* < 0.01). Estimated treatment differences were seen as early as 5 weeks, with 15 mg of tirzepatide to 1 mg of semaglutide showing −0.7 kg (95% CI: −1.4, −0.1) (*p* = 0.029). This trend widened at 28 weeks, with an estimated treatment difference of −4.3 kg (95% CI: −6.8 to −1.9) (*p* < 0.001). Fat and fat‐free mass significantly decreased for both semaglutide and tirzepatide (*p* < 0.001) as well. Fat mass and fat‐free mass reductions with tirzepatide compared to semaglutide were −3.8 kg (95% CI: −6.2 to −1.4, *p* = 0.02) and −0.8 kg (95% CI: −1.5 to −0.1, *p* = 0.018), respectively. Interestingly, it was found that both tirzepatide and semaglutide groups had significantly reduced appetite compared to baseline (*p* < 0.001), with the tirzepatide group also showing a significant difference in appetite compared to the placebo group (*p* = 0.007) but not semaglutide (*p* = 0.260) [10].

Rodriguez et al. reported changes in body weight over 12 months between the 5 mg tirzepatide group (9193 patients) and the 0.5 mg semaglutide group (9192 patients) [5]. The authors used brand as a proxy for the target dose, although they recognised that patients in both groups may have received higher or lower doses than the standard doses. For proportions of weight loss such as body weight loss of > 5%, > 10% and > 15%, tirzepatide had 81.8%, 62.1% and 42.3% achieving these endpoints compared to 66.5%, 37.1% and 18.1% for semaglutide. Hazard ratios comparing the tirzepatide with the semaglutide groups were 1.76, 2.54 and 3.24 for 5%, 10% and 15% or more weight loss, respectively [5]. It is important to note that patients with tirzepatide were younger with a greater proportion that was female, White and had a college education compared to semaglutide. However, the baseline weight was similar between groups.

In another study, Anson et al. evaluated weight loss at one year in patients without T2DM and found a significantly decreased body weight for tirzepatide at −7.7 kg (*p* < 0.001) and semaglutide at −4.8 kg (*p* < 0.001) [11]. It was noted that, due to the real‐world nature of the data, accurate drug dosages that were prescribed or the number of doses each participant received was not able to be confirmed.

### 3.3 Adverse Events

Adverse events (AEs) were reported in four studies, with the most common being gastrointestinal (GI) AEs [5, 9, 10, 11]. Across two studies, the total number of people who experienced at least one AE was 1354 (67.8%), with 988 (68.0%) people in the tirzepatide group and 344 (67.1%) people in the semaglutide group [9, 10]. 966 (48.4%) people experienced GI AEs, with 771 (53.0%) in the tirzepatide group and 267 (52.0%) in the semaglutide group [9, 10]. Frias et al. reported that 66.3% of the patients experienced 1 or more events, with 5.2% experiencing one or more serious AEs. 13 deaths were noted in the study, with investigators ruling that none of the deaths were related to the tirzepatide or semaglutide interventions. It was noted that the AEs were more common with 10 and 15 mg tirzepatide compared to the 5 mg tirzepatide and semaglutide groups [9]. Rodriguez et al. reported GI AEs rate per 1000 person‐years, with no significant differences in AEs noted between the two groups [5]. For the non‐T2DM cohort, Anson et al. reported no statistically significant difference between the tirzepatide and semaglutide groups in clinically significant hypoglycaemia or acute pancreatitis rates. As for the T2DM cohort, compared to semaglutide, tirzepatide was associated with a significant decrease in all‐cause mortality over 12 months as well as cerebral infarction rates. The tirzepatide group also had a significant increase in diabetic neuropathy rates. No significant difference in rates was found for heart failure, ischemic heart disease, retinopathy, nephropathy, or suicidal attempt or ideation [11]. A summary of AEs is provided in Table 3.

**TABLE 3:** Adverse events for tirzepatide and semaglutide.

	Tirzepatide				Semaglutide			
	Frias 2021	Heise 2022	Rodriguez 2024^a^	Anson 2024	Frias 2021	Heise 2022	Rodriguez 2024^a^	Anson 2024
Total adverse events (dosages)	63.6% (5 mg) 68.7% (10 mg) 68.9% (15 mg)	96% (15 mg)	NR	NR	64.2% (1 mg)	98% (1 mg)	NR	NR
Total gastrointestinal	40% 46.1% 44.9%	NR	NR	NR	41.2%	NR	NR	NR
Nausea	17.4% 19.2% 22.1%	24%	NR	NR	17.9%	30%	NR	NR
Diarrhoea	13.2% 16.4% 13.8%	20%	NR	NR	11.5%	30%	NR	NR
Vomiting	5.7% 8.5% 9.8%	7%	NR	NR	8.3%	11%	NR	NR
Dyspepsia	7.2% 6.2% 9.1%	7%	NR	NR	6.6%	32%	NR	NR
Decreased appetite	7.4% 7.2% 8.9%	60%	NR	NR	5.3%	70%	NR	NR
Constipation	6.8% 4.5% 4.5%	13%	NR	NR	5.8%	18%	NR	NR
Abdominal Pain	3.0% 4.5% 5.1%	11%	NR	NR	5.1%	11%	NR	NR
Bowel obstruction	NR	NR	6.26%	NR	NR	NR	5.54%	NR
Cholecystitis	NR	NR	6.50%	NR	NR	NR	5.06%	NR
Cholelithiasis	NR	NR	11.89%	NR	NR	NR	12.66%	NR
Gastroenteritis	NR	NR	19.75%	NR	NR	NR	20.07%	NR
Gastroparesis	NR	NR	3.61%	NR	NR	NR	4.81%	NR
Pancreatitis	NR	NR	3.84%	NR	NR	NR	3.60%	NR
All‐cause mortality	NR	NR	NR	0.18%	NR	NR	NR	0.59%
Ischemic heart disease	NR	NR	NR	2.74%	NR	NR	NR	2.86%
Cerebral infarction	NR	NR	NR	0.34%	NR	NR	NR	0.78%
Heart failure	NR	NR	NR	0.82%	NR	NR	NR	1.19%
Acute coronary syndrome	NR	NR	NR	1.37%	NR	NR	NR	1.46%
Diabetic retinopathy	NR	NR	NR	6.20%	NR	NR	NR	5.59%
Diabetic nephropathy	NR	NR	NR	4.0%	NR	NR	NR	3.40%
Diabetic neuropathy	NR	NR	NR	17.86%	NR	NR	NR	15.62%
Suicidal attempt and ideation	NR	NR	NR	0.17%	NR	NR	NR	0.31%

^a^ Event rates per 1000 person‐years.

## 4 Discussion

This systematic review analysed four comparative studies (two RCTs/two non‐RCTs) between tirzepatide and semaglutide, with a total of 28,827 patients (14,870 tirzepatide/13,928 semaglutide), a mean age of 55.7 years and a mean follow‐up of 35.9 weeks. The main findings in this study were that tirzepatide demonstrated greater mean weight loss than semaglutide but with a greater rate of AEs, primarily GI AEs. The forest plot illustrates a range of effect sizes across studies, suggesting that differences in baseline characteristics and study methodologies contribute to the variability in weight loss outcomes between tirzepatide and semaglutide. The inclusion of both RCTs and observational studies may introduce methodological inconsistencies, as RCTs tend to have stricter inclusion criteria, whereas real‐world studies encompass more diverse populations. Additionally, differences in the titration protocols and treatment durations across studies likely affect the magnitude of weight loss observed. This level of heterogeneity suggests that while tirzepatide consistently shows greater weight loss than semaglutide, the magnitude of the effect may depend on specific patient or study characteristics. Furthermore, the doses of semaglutide used are lower than the approved doses for T2DM or obesity, which may not reflect the results seen at higher doses.

### 4.1 Weight Loss

Obesity is closely related to the lifetime risk for the development of T2DM in men, increasing from 7% to 70% with a BMI of less than 18.5 kg/m^2^ to greater than 35 kg/m^2^, respectively [12]. Similarly, the lifetime risk increases in females from 12% to 74% with the aforementioned BMI values. An estimated majority (~86%) of T2DM patients are also overweight or obese [13]. Notably, weight loss of 5%–10% has been shown to improve glycaemic control and decrease the incidence of diabetes by over 50% [12]. At a 7‐year follow‐up, bariatric surgery elicited an 18.2% diabetes remission rate compared to the medical/lifestyle group at 6.2% (*p* = 0.02) [14]. However, bariatric surgery is considered a last resort if lifestyle modifications or medical therapies fail. GLP‐1 RAs (liraglutide, semaglutide, tirzepatide) have been approved to treat obesity and provide these effects via increased insulin release, increased insulin sensitivity, decreased gastric emptying and acting on central receptors in the central nervous system (CNS) to reduce appetite [12]. GLP‐1 RA's effects extend beyond T2DM and obesity and into reducing the risk of systemic conditions as well [15]. Nassar et al. conducted a retrospective cohort study with data spanning more than 5‐year follow‐up period with patients with T2DM or obesity. GLP‐1 RA treatment was found to significantly lower incidences of dementia (risk difference (RD): −0.010), Alzheimer's disease (RD: −0.003), Parkinson's disease (RD: −0.002), pancreatic cancer (RD: −0.003), systemic lupus erythematosus (RD: −0.001) and systemic sclerosis (RD: −0.000) with *p* < 0.001 for all. However, bronchial asthma had a slight increase (RD: 0.002, *p* < 0.001) [15].

In the direct comparative studies analysed in this meta‐analysis, tirzepatide demonstrated superior weight loss compared to semaglutide, supporting the notion of enhanced clinical efficacy of dual GLP‐1 and GIP agonism. The efficacy of semaglutide has been established throughout the SUSTAIN trials, where semaglutide showed significantly greater weight loss compared to placebo and active comparators (*p* < 0.0001 for all) [16]. Semaglutide also achieved greater proportions of weight loss over 5% or 10% (*p* < 0.0001 for all) [16]. Additionally, the STEP trials noted that semaglutide 2.4 mg had an average weight loss reduction of up to 16% [17]. Tirzepatide has also been established to be efficacious in reducing body weight, with up to 20.9% weight loss seen across the SURMOUNT trials [18, 19, 20, 21]. However, it is important to note that these trials evaluated each of these GLP‐1 RAs independently rather than direct head‐to‐head trials. Thus, the data in this meta‐analysis provide more clinically relevant comparisons between these agents.

Furthermore, the interaction between lifestyle intervention and usage of GLP‐1 RAs is still unclear. However, Wadden et al. suggest that an initial lifestyle intervention period followed by medication addition can maximise weight reduction [20]. This stems from the observation that STEP‐1 (semaglutide only) and STEP‐3 (lifestyle and semaglutide) did not produce an additive benefit towards weight loss [17]. These findings in the literature combined with this study's results suggest that adding semaglutide or tirzepatide produces clinically significant weight loss that can be further potentiated through diet and exercise habit changes. There also appears to be a dose‐dependent improvement in clinical outcomes (glycemic control and weight loss) with tolerable safety profiles at higher doses [9, 16, 22].

### 4.2 Adverse Events

Due to the limited amount of head‐to‐head trials, a comprehensive comparison of AEs between semaglutide and tirzepatide was unable to be done. Of the included studies, semaglutide and tirzepatide had similar GI AE profiles and tolerability, which were dose‐dependent. The monotherapy trials for semaglutide and tirzepatide can provide insight into the tolerability of these agents. Semaglutide in the SUSTAIN trials exhibited similar or greater rates of AEs, with the most common system affected being GI. However, the rates of severe AEs were comparable between all treatment arms. Semaglutide's GI AEs ranged from 27% to 44% compared to 15% with placebo and 48% with dulaglutide. Most AEs experienced by GLP‐1 RA cohorts were transient and generally categorised as mild or moderate [16]. Notably, the proportion of patients who discontinued treatment due to AEs was higher with semaglutide compared to its comparators (5% to 14% vs. 1% to 8%) [16]. A pooled analysis of 16 phase IIIa RCTs from the SUSTAIN and PIONEER trials (11,159 patients) found that 39.1% to 41.9% of subcutaneous and oral semaglutide, respectively, reported GI AEs compared to 22.0% to 24.8% with its comparators [23]. However, the tolerance of semaglutide is considered generally similar to other GLP‐1 RAs. In the SURMOUNT‐1 trial, the most common AE for tirzepatide was also GI of mild and moderate severity. The treatment discontinuation proportions for 5 mg, 10 mg and 15 mg were 4.3%, 7.1% and 6.2%, respectively [18]. SURMOUNT‐2 also found GI AEs to be the most commonly reported AE, with less than 5% of AEs leading to treatment discontinuation [19]. In obesity patients in SURMOUNT‐3 and SURMOUNT‐4, a high rate of AEs of 87.1% and 81.0%, respectively, was observed, also with GI AEs being the most frequent system affected. A recent meta‐analysis of 10 tirzepatide trials (*n* = 6836) found a dose‐dependent relationship for GI AEs, with 5 mg, 10 mg and 15 mg reporting 39% (95% CI: 35%–43%), 46% (95% CI: 42%–49%) and 49% (95% CI: 38%–60%), respectively [24]. Furthermore, the risk and recurrence of acute pancreatitis is a serious health concern for patients taking GLP‐1 RAs. Nassar et al. conducted a retrospective study comparing the recurrence rates of acute pancreatitis among patients with T2DM or obesity taking GLP‐1 RAs versus those who did not [25]. Overall, GLP‐1 RA users had significantly lower recurrence rates of acute pancreatitis at 13.8% versus 40.9% for non‐GLP‐1 RA users. Additionally, semaglutide and tirzepatide had the lowest recurrence risks, with the former reporting 11.7% and the latter displaying a significantly lower rate at 6.2% [25].

An important note for GLP‐1 GI AEs is that standard approaches may not correctly characterise the incidence of GI AEs given the mechanism of action of GLP‐1 RAs, which decrease gastric emptying and suppress appetite to reduce overall food intake. Rather than vomiting, nausea was reported more frequently as the reason for treatment discontinuation in the SUSTAIN and PIONEER trials [23]. However, a gradual dose escalation may be able to increase tolerability and reduce the frequency of GI AEs [22]. There has been some speculation on the contribution of GI AEs to the weight reduction seen in GLP‐1 RAs, but mediation analysis suggests a minimal contribution [26]. Overall, both semaglutide and tirzepatide are well tolerated and AEs are deemed mild to moderate in severity.

## 5 Implications

The substantial weight loss induced by semaglutide and tirzepatide provides an attractive modality for patients on their weight loss journey. Their physiological mechanism of action in reducing overall food intake may allow patients to make changes to their diets with greater ease compared to traditional lifestyle interventions and counselling. Weight loss can also lead to improved physical activity, an important lifestyle habit that can further improve cardiovascular health. In addition to the effects of weight loss on attenuating risk for cardiovascular disease, semaglutide also demonstrates cardiovascular safety and has recently been approved in March 2024 by the FDA for reducing cardiovascular mortality [27]. It is important to note that this cardiovascular benefit is not observed with oral semaglutide [27]. Ultimately, these results for semaglutide and tirzepatide emphasise the need to ensure the maintenance of weight loss regardless of which medication an individual takes.

Future studies should focus on conducting longer‐term follow‐up studies to better elucidate the weight loss maintenance and safety profiles of these medications. High‐quality studies with standardised baseline patient demographics are also important, as this may affect the outcomes seen. Additionally, more head‐to‐head studies will better characterise the effect of one GLP‐1 RA over another. A focus on weight loss maintenance should also be analysed due to the possibility of weight regain after cessation of these medications [21].

## 6 Limitations

The results from this study must be analysed within the context of its limitations. First, there is a relative paucity in head‐to‐head trials comparing tirzepatide against semaglutide. Further studies, especially with longer‐duration trials and varying dosages, can better elucidate the long‐term weight loss reduction and maintenance and characterise the safety profile over time. Second, two non‐RCTs were included in this study, which may introduce bias that can affect the meta‐analysis results. Third, there was heterogeneity across the patient characteristics and the dose regimens in these studies. Fourth, the semaglutide dosage used across several studies, as noted earlier, is lower than the approved dose for T2DM and obesity, as mentioned earlier, which may not fully reflect the results seen at clinically relevant doses.

## 7 Conclusion

Both tirzepatide and semaglutide demonstrated considerable efficacy in reducing body weight with minimal to moderate severity AEs. However, tirzepatide consistently produced greater weight loss compared to semaglutide but with higher rates of AEs. The limited number of direct comparative studies and relatively short follow‐up periods prevent a definitive conclusion of tirzepatide's superiority over semaglutide. Future research should focus on larger, long‐term, head‐to‐head trials to confirm these findings and provide more robust data on the comparative effectiveness of these medications.

## Author Contributions

J.W.: conceptualization, data curation, formal analysis, investigation, methodology, project administration, resources, software, supervision, validation, visualization, writing – original draft, writing – review and editing. B.S.: conceptualization, data curation, formal analysis, investigation, methodology, validation, visualization, writing – original draft, writing – review and editing. D.N.: conceptualization, data curation, formal analysis, investigation, methodology, validation, visualization, writing – original draft, writing – review and editing. C.H.V.: conceptualization, data curation, formal analysis, validation, visualization, writing – original draft, writing – review and editing. E.B.: conceptualization, data curation, formal analysis, validation, visualization, writing – original draft, writing – review and editing. A.T.: conceptualization, data curation, formal analysis, validation, visualization, writing – original draft, writing – review and editing. M.A.: conceptualization, data curation, formal analysis, validation, visualization, writing – review and editing. A.R.: data curation, formal analysis, validation, visualization, writing – review and editing. J.P.: data curation, formal analysis, resources, software, writing – review and editing. E.F.: conceptualization, investigation, methodology, project administration, resources, supervision, writing – review and editing.

## Conflicts of Interest

The authors declare no conflicts of interest.

## Supporting information


Table S1

Table S2

## Data Availability

The datasets used and/or analysed in the current study are available upon reasonable request. Please contact J.W. to request data from the study.
