# Supplementary material for: Tirzepatide Versus Semaglutide on Weight Loss in Type 2 Diabetes Patients: A Systematic Review and Meta‐Analysis of Direct Comparative Studies
Source: Endocrinol Diabetes Metab. 2025 Apr 4;8(3):e70045. doi: 10.1002/edm2.70045 (PMC11970626; doi:10.1002/edm2.70045)
Supplement: Supplementary file 1 — Table S1 [file EDM2-8-e70045-s001.docx]

| **Author** | **Sequence Generation** | **Allocation Concealment** | **Blinding of Participants and Personnel** | **Blinding of Outcome Assessors** | **Incomplete Outcome Data** | **Selective Outcome Reporting** | **Other Sources of Bias** |
| --- | --- | --- | --- | --- | --- | --- | --- |
| Frias 2021 | **Low** | **Low** | **High** | **Low** | **Low** | **Low** | **Unsure** |
| Heise 2022 | **Low** | **Low** | **Low** | **Low** | **Low** | **Low** | **Low** |

**Table S1: Cochrane Risk of Bias Tool for Included Studies**
