# Supplementary material for: Tirzepatide Versus Semaglutide on Weight Loss in Type 2 Diabetes Patients: A Systematic Review and Meta‐Analysis of Direct Comparative Studies
Source: Endocrinol Diabetes Metab. 2025 Apr 4;8(3):e70045. doi: 10.1002/edm2.70045 (PMC11970626; doi:10.1002/edm2.70045)
Supplement: Supplementary file 2 — Table S2 [file EDM2-8-e70045-s002.docx]

| **Author** | **Clearly stated aim** | **Inclusion of consecutive patients** | **Prospective collection of data** | **Endpoints appropriate to the aim of the study** | **Unbiased assessment of the study endpoint** | **Follow-up period appropriate to the aim of the study** | **Loss to follow up less than 5%** | **Prospective calculation of the study size** | **An adequate control group** | **Contemporary groups** | **Baseline equivalence of groups** | **Adequate statistical analyses** | **Total** |
| --- | --- | --- | --- | --- | --- | --- | --- | --- | --- | --- | --- | --- | --- |
| Frias 2021 | **2** | **2** | **2** | **2** | **2** | **2** | **2** | **2** | **0** | **0** | **2** | **2** | **20** |
| Heise 2022 | **2** | **1** | **2** | **2** | **2** | **2** | **2** | **2** | **2** | **2** | **2** | **2** | **23** |
| Zakaria 2024 | **2** | **1** | **2** | **2** | **2** | **2** | **2** | **2** | **-** | **-** | **-** | **-** | **15** |
| Rodriguez 2024 | **2** | **2** | **2** | **2** | **2** | **1** | **2** | **2** | **-** | **-** | **-** | **-** | **15** |
| Anson 2024 | **2** | **2** | **2** | **2** | **1** | **2** | **2** | **2** | **-** | **-** | **-** | **-** | **15** |

**Table S2: Methodological Index for Non-Randomized Studies (MINORS) for Included Studies**
